# A Review on Music Interventions for Frontotemporal Aphasia and a Proposal for Alternative Treatments

**DOI:** 10.3390/biomedicines11010084

**Published:** 2022-12-29

**Authors:** Vittoria Spinosa, Alessandra Vitulli, Giancarlo Logroscino, Elvira Brattico

**Affiliations:** 1 Center for Neurodegenerative Diseases and the Aging Brain, Department of Clinical Research in Neurology, University of Bari “Aldo Moro”, Pia Fondazione Cardinale G. Panico, 73039 Tricase, Italy; 2 Department of Basic Medicine, Neuroscience, and Sense Organs, University of Bari “Aldo Moro”, 70121 Bari, Italy; 3 Center for Music in the Brain, Department of Clinical Medicine, Aarhus University, 8000 Aarhus, Denmark; 4 Department of Education, Psychology, Communication, University of Bari “Aldo Moro”, 70121 Bari, Italy

**Keywords:** music, rhythm, language, primary progressive aphasia, intervention, frontotemporal dementia

## Abstract

Frontotemporal dementia (FTD) is a rare neurodegenerative disease, characterized by behavioral and language impairments. Primary progressive aphasia (PPA) is the linguistic variant of this heterogeneous disorder. To date, there is a lack of consensus about which interventions are effective in these patients. However, several studies show that music-based interventions are beneficial in neurological diseases. This study aims, primarily, to establish the state of the art of music-based interventions designed for PPA due to FTD and, secondarily, to inform the planning of PPA-dedicated future interventions for Italian neurological institutions. The first aim is fulfilled by a review which critically screens the neurological studies examining the effects of music- and/or rhythm-based interventions, especially, on language rehabilitation in aphasic FTD. We found that only two papers fulfilled our criteria and concerned specifically aphasic patients due to FTD. Of those, one paper reported a study conducted in an Italian institution. Most of the reviewed studies focused, instead, on aphasia in post-stroke patients. The results of our review invite further studies to investigate the role of music as a valuable support in the therapy for neurodegenerative patients with language problems and in particular to PPA due to FTD. Moreover, based on this initial work, we can delineate new music-based interventions dedicated to PPA for Italian institutions.

## 1. Introduction

Frontotemporal dementia (FTD) is a neurodegenerative disease that is a result of the progressive atrophy of the frontal and temporal lobes, and can be associated with several genes [1,2]. The FTD spectrum is associated with language, behavioral, and motor phenotypes and includes different clinical syndromes which could include FTD-motor neuronal disease, progressive supranuclear palsy (PSP), and corticobasal syndrome (CBS) [3,4].

A recent paper reported that FTD could be considered a rare disease [5]. Indeed, this study conducted in two administrative districts from the south (province of Lecce) and from the north (province of Brescia) of Italy, with about two million inhabitants, in 2017, found 63 cases of FTD, including patients with FTD and its different clinical syndromes. Thus, the incidence was 3.05 per 100,000 person-years (py). In Europe, its incidence is still unclear. The “FRONTotemporal dementia Incidence European Research Study” (FRONTIERS) was designed to examine the incidence of this FTD at a wider level, with the intention of improving the FTD treatment strategies in public health [3].

Overall, FTD is characterized by disturbances in psychosocial and linguistic areas [6]. Indeed, the FTD is represented by two main phenotypes: the behavioral (bvFTD), and the linguistic variant that is the primary progressive aphasia (PPA).

PPA is a neurodegenerative disorder which selectively impacts the language domain [7,8]. A progressive language disorder was first described by Pick [9] and Serieux [10]. In 1982, Mesulam and colleagues [11] described six cases of slowly progressing aphasic disorder without the typical disturbances of dementia. In 2003, Mesulam [12] defined the criteria of PPA, specifying that, at the onset, language must be the prominent affected function at least for two years. However, during the progression of the disorder, other cognitive functions may be impaired, but language must be more affected through the course of the disease. No stroke or tumors must be the cause of the aphasia [12]. Recently, the classification by Gorno-Tempini of the different variants of PPA has played an important role in the recognition of this language disease as a neurodegenerative disease [13]. They proposed a classification to better subdivide PPA into three main groups: the semantic, nonfluent/agrammatic, and logopenic variants. The underlying neuropathology of the nonfluent/agrammatic (nfvPPA) and semantic variants (svPPA) is FTD, whereas the logopenic variant (lvPPA) is more frequently found in Alzeimer’s disease (AD) [5,7].

Currently there are no drugs that are effective in treating FTD [4]. Speech and language therapists have used some speech therapy exercises and strategies on PPA patients that have been developed for other forms of language disorders, particularly for post-stroke aphasia [14]. However, the literature regarding the efficacy of interventions focused on PPA is not so robust [15]. The study by Henry and colleagues [16] examined, for the first time, the efficacy of script training on the nfvPPA variant. Specifically, this training, named VISTA (video-implemented script training for aphasia), was conducted on site by a clinician and at home through video stimuli in which patients could replicate attempt each script that was produced by a healthy speaker. The authors found improvements in these patients.

Other types of interventions for PPA have focused on the written or oral naming of target items [17], and are administered either in person or via teletherapy. Particularly, “word retrieval training” makes patients work on producing spoken and written names of personally relevant target items using a self-cueing hierarchy. This treatment aims to utilize all the strategies capitalizing on spared cognitive-linguistic abilities to support word retrieval. Typically, the sessions each occur twice a week, last one hour, and involve a clinician. Daily home practice exercises are also included.

In recent years, studies investigating the effect of transcranial direct current stimulation (tDCS) or of transcranial magnetic stimulation (TMS) on PPA are also emerging, but further studies on long-term efficacy are still needed [18,19].

In the field of neurological rehabilitation, alternative therapeutic approaches are emerging with accumulating empirical evidence. Among the most efficacious there is the music-based intervention, thus termed when the rehabilitative exercises and practices are based on melodic or rhythmic activities, or music therapy (or neurology-based music therapy [20]), thus termed when a professional music therapist is involved. Music-based interventions and therapy have been consistently successful in cognitive and language rehabilitation of post-stroke aphasia [21,22,23,24]. Several systematic reviews and meta-analyses do exist in the literature that testify to the small to medium effects of music-based interventions on several neurological and psychological symptoms in stroke and dementia [25,26,27,28,29,30]. One of the most common music interventions in post-stroke aphasia is Melodic Intonation Therapy (MIT) [31,32,33]. This language therapy, developed in 1973, consists of music- and rhythm-based rehabilitation. Specifically, the subject is invited to intonate single words or sentences in order to facilitate, in the final step, their vocalization. In addition, the therapist invites the subject to follow a specific rhythm with the left hand in order to facilitate language rehabilitation. Initially, the therapist guides the subject, until they reach an autonomous speech production. However, it is still in debate as to which brain mechanisms underly this therapy and which are the specific elements that are beneficial for neurorehabilitation [34]. Two recent systematic reviews and meta-analyses focusing on post-stroke patients showed that MIT is beneficial to language functions [35], specifically to functional communication and repetition tasks [36]. Zumbansen and colleagues [37] suggest that MIT is more efficient in apraxia of speech (AoS), which is a speech disorder that can be defined as a deficit in motor planning [38,39,40]. Moreover, so far three main protocols have been identified that are related to MIT: the original version of MIT, the ‘French thérapie mélodique et rythmée’ (TMR), and other ‘palliative versions of MIT’ [37]. An Italian adaptation of TMR, ‘Melodic Rhythmic Therapy’ (RMT), was proposed by Cortese and collaborators [41].

Music might be an alternative route to improving language brain functions at different disease stages, due to several reasons: first, music perception and production can rely on shared neural resources with language [42], second, music is highly motivating and engaging [43], and third, residual musical skills do exist even in highly impaired patients [33]. Music-based interventions can be subdivided into active and passive, whether the subject is engaged in singing, playing or clapping, or whether the subject is invited only to listen to music. Moreover, independently of being engaged in individual or group sessions, music has the power to create social interaction [44]. Differently to AD patients, FTD is mainly characterized by difficulties in emotional processing during social interactions [45]. Since it has been shown that the music valence affects the perception of the body expression, we could speculate that music could also have an impact on social cognition in FTD patients [46].

Several studies have shown that language is often impaired in the early stages of AD [47,48,49,50], and musical interventions for AD have also been described as non-pharmacological treatment for its cognitive and psychological symptoms [51]. Despite there being studies showing the efficacy of music therapy on FTD as a treatment for cognitive, psychological, and behavioral disorders [52,53], studies that specifically address the use of music as a treatment for aphasia in frontotemporal dementia seem to be missing. However, Grube and collaborators [54] have shown that pitch, rhythm, and timbre processing differ based on the type of PPA aphasia. Indeed, nfvPPA patients seemed to be the patients with the greatest impairment, with an impairment in the acoustic, linguistic, and affective components of prosody [55]. Sv-PPA variant patients were also impaired, albeit to a lesser extent, while lvPPA patients have shown no notable impairment [54]. This could prove that there is a central auditory deficit for non-linguistic stimuli in the perceptual processing of tone sequences [54]. However, a study by Maruta and colleagues [56] showed how delayed auditory feedback can cause effects similar to nonfluent aphasia in healthy subjects, suggesting different pathways of language and nonverbal sounds in reception and production. Moreover, Goll and colleagues (2010) examined the processing of nonverbal sounds in FTD patients with nfv- and sv-PPA through a neuropsychological battery. These aphasic patients showed nonverbal sound analysis deficits compared to age-matched individuals with differences only between nonfluent and semantic aphasia. Another study by Goll and collaborators [57], including patients with AD and lvPPA, has shown how the profile differs in each dementia syndrome. Finally, a study by Maruta and colleagues [56] showed how delayed auditory feedback can cause effects similar to nonfluent aphasia in healthy subjects, suggesting different pathways of language and nonverbal sounds in reception and production.

Given the small number of intervention studies on PPA compared to studies on post-stroke, and given the efficacy highlighted in music interventions for the latter, e.g., [21,58,59,60,61], we would like to, first, critically screen the literature to search for any PPA-dedicated studies on music interventions and then verify if there is efficacy in any music-based treatment also for PPAs associated with FTD.

However, a systematic strategy for music interventions designed specifically for PPA seems to still be absent in the literature and in clinical practices. Hence, this work includes, first, a review aimed at finding answers to the following questions: are there studies investigating the effects of music interventions on FTD aphasia? Second, the study wishes to inform the planning of PPA-dedicated music interventions for Italian neurological institutions.

Hence, here we wished to focus on PPA to analyze, by means of a review, state-of-the-art music-based interventions. This analysis has the secondary scope to examine music-based intervention studies on PPA due to FTD carried out in Italy, and to propose innovative and personalized strategies against FTD aphasia.

## 2. Materials and Methods

### 2.1. Literature Search and Study Eligibility

Initially, we applied a systematic review methodology following the PRISMA statement [62]. The protocol for this systematic review was registered on PROSPERO (identity number, 367559). A systematic search was carried out in May 2022 using PubMed to identify studies investigating aphasia patients with neurodegenerative diseases due to FTD. We used the following keywords: aphasia, frontotemporal dementia, randomized controlled trial, intervention, stimulation, rehabilitation, training, therapy, approach, rate, rhythm, melodic, melody, and music. We report the full string used for this search as follows:

(“aphasia” [All Fields] OR “frontotemporal dementia” [All Fields]) AND (“randomized controlled trial” [All Fields] OR “clinical trial” [All Fields] OR “intervention” [All Fields] OR “stimulation” [All Fields] OR “rehabilitation” [All Fields] OR “training” [All Fields] OR “therapy” [All Fields] OR “approach” [All Fields]) AND (“rate” [All Fields] “rhythm” [All Fields] OR “melodic” [All Fields] OR “melody” [All Fields] OR “music” [All Fields])

Studies were included if they met the following criteria: (1) patients with aphasia due to frontotemporal dementia; (2) studies using musical interventions; (3) adult participants aged over 18. The following exclusion criteria were used to select studies of interest: (1) not peer-reviewed studies; (2) studies in languages other than English; (3) reviews; (4) meta-analyses; (5) animal studies; (6) post-mortem studies; (7) study participants with neurodevelopmental disorders; (8) aphasia due to stroke or trauma and patients with tumor; (9) studies published before 1980; (10) non journal articles (e.g., only abstracts or non-available full-text).

After conducting the literature search, the studies were screened independently by two researchers based on titles and abstracts. Discrepancies were discussed with a third researcher. Subsequently, the full-texts were retrieved and evaluated. Finally, a table was created to extract the most relevant data, and to assess the methodological quality of the included studies. The exiguous number of studies, resulting from the search, impeded the proper completion of the systematic review process. Thus, we finally critically examined the two resulting studies.

### 2.2. Data Extraction

The following variables were extracted from each study: general information about the study (authors, article title, year of publication, type of publication, and geographic location); study characteristics (objectives, study inclusion and exclusion criteria); participant characteristics (sample size, mean age, gender, education, and disease duration); overall outcome data/results (main outcomes, type of analysis, number of participants enrolled, and number of participants included in the analysis); assessment (language assessment, neuropsychological assessment); and details on the intervention (type, duration, focus, and responsible).

## 3. Results

**Figure 1:**
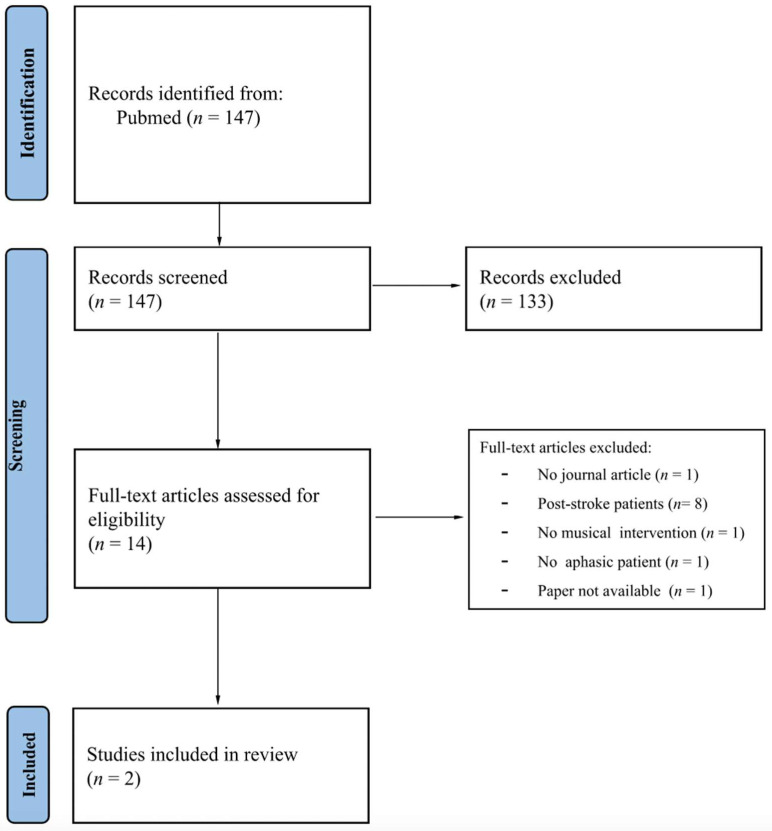
Modified version of the PRISMA diagram flow of the review.

The initial literature search of keywords produced 147 results. Due to the inclusion criteria, 133 studies were excluded. Among the 14 studies screened for eligibility, two met our criteria. Supplementary Materials contains the full list of included and excluded studies. A flow chart summarizing the selection process is depicted in Figure 1.

Most of the studies were excluded because they were not focused on neurodegenerative patients. Indeed, we found that 79 studies were on post-stroke patients and 1 on neurodevelopmental disorders. Moreover, we found 13 studies lacking interventions designed for aphasia patients. There were 33 reviews and 1 meta-analysis. Five studies were published before 1980. Fifteen studies were in a language different from English. Table 1 provides a list of the excluded full-text papers and the related reason.

### 3.1. Study Characteristics

As visible from Table 2, the studies included in this review were only 2 case studies. Beber and colleagues [75] focused on a patient with frontotemporal diagnosis having global aphasia, whilst Raglio and colleagues [76] focused on a patient diagnosed with nfvPPA. However, no details were provided about the onset of their symptoms, nor on the onset of the aphasia. The interventions were briefly outlined in the manuscript and are summarized in the sections below.

#### 3.1.1. Sociodemographic Variables

The age of the subjects was specified in each study. The mean age of the participant was 65 years old. As concerning education, one participant was illiterate [75], whilst no information was provided about the education of the other participant [76].

Geographically, one study was conducted in Brazil [75] and the other one in Italy [76]. However, the nationality of the subject was not specified.

#### 3.1.2. Assessment and Treatments

Cognitive screening. Both studies used the Mini-Mental State Examination (MMSE) as cognitive screening. However, the case described by Raglio and colleagues [76] was not able to answer the questions in this screening. In addition to the MMSE, Beber and colleagues [75] used the Clinical Rating Scale (CDR), whilst Raglio and colleagues [76] deeply evaluated the behavior through the Neuropsychiatric Inventory (NPI), the Cohen Mansfield Agitation Inventory (CMAI), and the Cornell Scale for Depression in Dementia (CSDD).

Speech assessment. In the study by Beber and colleagues [75], motor-speech was assessed with a qualitative instrument. The verbal apraxia assessment included repetition of words and sentences, emission of automatisms, spontaneous speech, and reading aloud. Oro-facial structure movements were also evaluated in order to assess the nonverbal apraxia.

Language assessment. The patient visited by Beber and colleagues [75] was assessed with an informal spontaneous evaluation and the Brazilian Montreal Toulouse Language Assessment (MTL-BR).

State Pre-Intervention. The patient described by Beber and colleagues [75] presented with difficulty in starting speech, with blocks and sound repetitions. Moreover, as well as nonverbal difficulties, she had lips, tongue, and facial incoordination.

Type, frequency, and duration of intervention. Both studies used an active intervention. However, in one study a speech therapist conducted a speech and language therapy based on rhythm [75]. The main parts of this intervention were as follows: lengthen the first sound, emit syllables following the rhythm provided by the therapist, follow the rhythm of each word. The intervention lasted 45 min and was conducted once per week. Moreover, the therapist indicated some home-assignments to be done once per day with her husband, who had an active role. The intervention lasted 2 months. In the study by Raglio [76], a music therapist chose a music-therapy approach, based on the use of musical instruments and voice. No further details are provided in the article. This music-therapy intervention lasted 30 min and was articulated in 50 individual sessions, twice a week over 6 months.

State Post-Intervention. Post-intervention assessment was not conducted, because the patient was unable to attend, thus it was only qualitative and indirect [75]. However, after the intervention the patient was able to autonomously follow the learnt strategies, producing single words and short sentences, despite the apraxia of speech still being present. Raglio and colleagues [76] evaluated their patient at baseline, before treatment, after 25 sessions, at the end of treatment, and at 1-month after treatment through the same pretreatment scales. A qualitative assessment was conducted to evaluate the MT sessions. After the intervention, there was a significant decrease in the behavioral disturbances and in vocal production, indeed the vocalizations allowed the patient to communicate.

## 4. Discussion

Initially, we intended to conduct a systematic review, following PRISMA guidelines; however, the exiguous number of studies resulting from the search impeded the proper completion of a systematic review process. The review aimed at screening state-of-the-art music-based interventions for aphasic patients with FTD, and then critically analyzing all the studies, for the planning of future intervention studies to improve rehabilitative approaches in Italian institutions and beyond.

Our results evidence that, in contrast to such a mass of music-based interventions and music therapy for stroke patients and for the alleviation of behavioral symptoms in dementia patients, music-based interventions dedicated to aphasic FTD seem to have not thus far attracted the attention of researchers. Indeed, the vast majority of studies were focused on aphasic patients due to stroke.

Moreover, there were few empirical studies, and this result suggests the difficulty of carrying out studies on FTD aphasic patients. This could be due to the low incidence of this disease, indeed, in 2019, Logroscino and collaborators [5] found, in an Italian cohort of 63 FTD patients, that only 19 patients were nfvPPA (30.16%, 95% CI 19.23–43.02%), whilst there were no svPPA.

Specifically, the two included studies were single-case studies. These studies differ in their goals, in their selection of clinical cases, in the type of intervention, in the therapist (speech vs. music therapist), and in the type and frequency of the assessments. Indeed, the objective in the study by Raglio and colleagues [76] was to treat the FTD patient globally, focusing both on behavioral and on linguistic disturbances, whilst the objective in the study by Beber and collaborators [75] was to treat the apraxia of speech, through an approach similar to the MIT. Both studies reported only qualitative results related to language.

Our results seem to be in line with the current literature. Indeed, in 2013, Carthery-Goulart and collaborators [15] showed that there were few studies and many of them were case reports. The small portion of subjects referred to specialized speech and language therapists could be due to a lack of robust research results [77]. Despite the low number of included studies, this review can help in examining many critical points with the aim of improving the quality of the interventions for aphasic FTD patients. 

Analyzing the potential reasons for the different amounts of studies focusing on post-stroke patients compared to those with PPA, we believe that one reason could be associated with the starting stage of the therapy. Indeed, most of the post-stroke patients usually pass for a rehabilitation process right after the stroke, while this is not valid for the PPA patients who begin the therapy at different disease stages and often do not arrive at the therapists during the early stages of the disease [77]. The spectrum of frontotemporal dementia is heterogeneous as well as the characteristics of the PPA. This impedes having empirical studies with a sufficient number of subjects per variant, diminishing the statistical power of their results, since the rehabilitative approach varies based on the specific variant. Indeed, there are different intervention studies on svPPA [78,79] and on nfvPPA [16,80].

Moreover, the study by Volkmer and collaborators [14] suggested the absence of a PPA-dedicated training for speech and language therapists. However, it is fundamental to have a deep knowledge of the specific tools to assess and to intervene on the PPA patient. Thus, it could be necessary to implement a specific training for therapists aimed at the treatment of PPA.

Considering the dramatically progressive trend of PPA, it is necessary to set up a flexible treatment plan that also meets the needs of the individual and the discomfort of the family members [14], modifying step by step the treatment based on the evolution of the aphasic clinical picture. A more robust collaboration between researchers and speech and language therapists could be useful to determine what is more relevant to each patient according to the knowledge of the experienced practitioner (practice-based evidence) [14].

Nowadays, the literature shows that music-based interventions are valid for post-stroke aphasia. However, the specific features of the aphasia that benefit from this treatment are still unclear. Considering that the AoS could be present both in nfvPPA and post-stroke patients, and that MIT seems to be beneficial specifically to AoS, then we could speculate that MIT could also support nfvPPA patients. Further studies on FTD-related PPA are necessary to better examine the disruptive mechanisms underlying PPA, in order to produce effective non-pharmacological therapies.

Further studies could be necessary to understand if passive musical intervention can also be used in very compromised patients, in the most advanced state of the disease, to alleviate depressive symptoms and feelings of discomfort. Indeed, music is a low-cost tool, based on a universal language which could be used widely in a standardized manner. The evidence of an association between prosody and music perception in healthy subjects seems to be more evident in the rhythm domain [81]. Thus, a rhythm-based assessment could be useful to compare aphasic patients who speak different languages. This would also facilitate the comparison of the results from different countries, providing more robust statistical power.

In Italy, the interest in PPA and in music-based approaches designed for neurodegenerative disorders is growing more and more. Among the studies carried out in Italy, some of them focused on PPA [82,83,84,85,86,87,88], while others focused on music-based rehabilitation and specifically on Alzheimer’s disease [89,90]. However, there is a lack of studies focusing on music-based interventions for FTD-related PPA.

As shown in our review, one out of two music-based intervention studies carried out on aphasia due to FTD is Italian [76]. Furthermore, there is an Italian study which has proposed an Italian version of the ‘French thérapie mélodique et rythmée’ (TMR) testing it on six post-stroke patients [41]. These results are encouraging for the future development of this type of approach in Italy.

MIT, the most popular music-based intervention for post-stroke aphasia, was developed based on the assumption and observation that severely compromised aphasic patients were able to sing folk songs with good articulation and this intervention was proposed when the others failed [33]. However, literature shows similarities and differences between PPA and post-stroke patients [91]. Thus, further studies are necessary to understand whether music can aid in rehabilitating common mechanisms across diseases. Overall, the collaboration between both music and speech-and-language therapists could be beneficial for the implementation of specific non-pharmacologic and universal tools for PPA due to FTD.

## 5. Conclusions

PPA is a neurodegenerative disease, characterized by a selective language impairment [7,8]. The results from our review support the broader existing evidence on the efficacy of music interventions for cognitive rehabilitation in stroke patients. However, so far, there are no robust results showing the efficacy of music interventions on PPA due to FTD, probably because of the rarity of the disease and because of the heterogeneity of the PPA variants. Thus, these results encourage the adaptation and the design of new intervention studies on PPA patients in order to investigate in a clinical controlled manner the effects of musical interventions on FTD patients with aphasia. Moreover, our findings illustrate the need for conducting wide music-intervention studies and developing language-independent music-based tools which could facilitate the sharing of results across countries. This would allow us to examine the language domain independently from the specific language spoken by the patient [92].

The comparison between these effects on aphasic post-stroke and FTD patients can provide useful information regarding these two diseases and can also contribute to study of the neuroanatomical structures of the brain mechanisms responsible for music and language.

## Figures and Tables

**Table 1:** Main characteristics of the studies excluded from this review.

No Journal Articles	Post-Stroke Patients	No Musical Intervention	No Aphasic Patient	Paper Not Available
Stahl et al., 2014 [63]	Jungblut et al., 2022 [64]	Wright et al., 2018 [65]	Brotons et al. [66]	Liechty, 2006 [67]
	Jungblut et al., 2014 [68]			
	Tomaino, 2012 [69]			
	Wambaugh et al., 2012 [70]			
	Stahl et al., 2011 [71]			
	Breier et al., 2010 [72]			
	Kim et al., 2008 [73]			
	Belin et al., 1996 [74]			

**Table 2:** Main characteristics of the two case studies included in the review.

Study	Disease	Age	Intervention	Responsible	Duration (m)	Frequency (per Week)
Beber et al. (2018) [75]	non-fluent PPA	72	rhythm-based on apraxia of speech	speech therapist	45	once
Raglio et al. (2012) [76]	FTD with global aphasia	58	active music-therapy	music therapist	30	twice

## Data Availability

Not applicable.

## Supplementary Materials

The following supporting information can be downloaded at: https://www.mdpi.com/article/10.3390/biomedicines11010084/s1. Full list of included and excluded studies, in CSV format, created with Rayyan software [93].
